# Significantly Reduced Intensity of Infection but Persistent Prevalence of Schistosomiasis in a Highly Endemic Region in Mali after Repeated Treatment

**DOI:** 10.1371/journal.pntd.0001774

**Published:** 2012-07-31

**Authors:** Aly Landouré, Robert Dembélé, Seydou Goita, Mamadou Kané, Marjon Tuinsma, Moussa Sacko, Emily Toubali, Michael D. French, Adama D. Keita, Alan Fenwick, Mamadou S. Traoré, Yaobi Zhang

**Affiliations:** 1 Institut National de Recherche en Santé Publique, BP 1771, Bamako, Mali; 2 Programme National de Lutte Contre les Schistosomiases et les Geohelminthes, Ministère de la Santé, Bamako, Mali; 3 Helen Keller International, Bamako, Mali; 4 Hôpital du Mali, Ministère de la Santé, Bamako, Mali; 5 Helen Keller International, New York, New York, United States of America; 6 Schistosomiasis Control Initiative, Imperial College London, London, United Kingdom; 7 Hôpital National du Point G, Centre Hospitalier Universitaire, Bamako, Mali; 8 Faculté de Médecine de Pharmacie et d'Odonto-Stomalogie, Bamako, Mali; 9 Helen Keller International, Regional Office for Africa, Dakar, Senegal; London School of Hygiene & Tropical Medicine, United Kingdom

## Abstract

**Background:**

Preventive chemotherapy against schistosomiasis has been implemented since 2005 in Mali, targeting school-age children and adults at high risk. A cross-sectional survey was conducted in 2010 to evaluate the impact of repeated treatment among school-age children in the highly-endemic region of Segou.

**Methodology/Principal Findings:**

The survey was conducted in six sentinel schools in three highly-endemic districts, and 640 school children aged 7–14 years were examined. Infections with *Schistosoma haematobium* and *S. mansoni* were diagnosed with the urine filtration and the Kato-Katz method respectively. Overall prevalence of *S. haematobium* infection was 61.7%, a significant reduction of 30% from the baseline in 2004 (p<0.01), while overall prevalence of *S. mansoni* infection was 12.7% which was not significantly different from the baseline. Overall mean intensity of *S. haematobium* and *S. mansoni* infection was 180.4 eggs/10 ml of urine and 88.2 epg in 2004 respectively. These were reduced to 33.2 eggs/10 ml of urine and 43.2 epg in 2010 respectively, a significant reduction of 81.6% and 51% (p<0.001). The proportion of heavy *S. haematobium* infections was reduced from 48.8% in 2004 to 13.8% in 2010, and the proportion of moderate and heavy *S. mansoni* infection was reduced from 15.6% in 2004 to 9.4% in 2010, both significantly (p<0.01). Mathematical modelling suggests that the observed results were in line with the expected changes.

**Conclusions/Significance:**

Significant reduction in intensity of infection on both infections and modest but significant reduction in *S. haematobium* prevalence were achieved in highly-endemic Segou region after repeated chemotherapy. However, persistent prevalence of both infections and relatively high level of intensity of *S. mansoni* infection suggest that more intensified control measures be implemented in order to achieve the goal of schistosomiasis elimination. In addition, closer monitoring and evaluation activities are needed in the programme to monitor the drug tolerance and to adjust treatment focus.

## Author Summary

Schistosomiasis or bilharzia is caused by infection with the trematode *Schistosoma* spp. It is widely prevalent in Mali, causing a major public health problem. One of the major control measures is mass treatment of population at risk, particularly school-age children, using the drug praziquantel. Mali started such mass drug treatments in 2005 and a cross-sectional survey was conducted in 2010 in highly endemic region Segou to evaluate the impact of the programme comparing with the baseline data collected in 2004. The repeated treatment significantly reduced the intensity of infection which is closely related to the morbidity of schistosomiasis, however, the prevalence persists with little reduction in the highly endemic region. The results suggest that more intensified control measures be implemented in order to achieve the goal of schistosomiasis elimination, possibly more frequent treatments, improved coverage and improved water and sanitation.

## Introduction

Schistosomiasis or bilharzia, caused by infection with *Schistosoma* spp, is one of the major neglected tropical diseases (NTDs). It remains a major cause of morbidity in developing countries, especially in sub-Saharan Africa. It has been estimated that around 200–209 million people worldwide may be infected with schistosomiasis and about 85% of these infections occur in Africa [1], [2], [3]. The mainstay of the current control strategy recommended by World Health Organization (WHO) against schistosomiasis is preventive chemotherapy (PCT) with praziquantel (PZQ) [4], [5]. The aim is to reduce morbidity due to schistosomiasis through regular treatment. Praziquantel is safe and efficacious against both *Schistosoma haematobium* (causing urogenital schistosomiasis) and *S. mansoni* (causing intestinal schistosomiasis), two major human species in sub-Saharan Africa. Repeated chemotherapy, usually as an annual regular treatment, can reduce infection and associated morbidity [6], [7], [8], [9]. WHO recently estimated that about 237 million people, including 109 million school-age children, globally require annual treatment for schistosomiasis, of which 220 million are in Africa, and only 12.7% of this estimated population have received treatment in 2010 [10].

In Mali, both urogenital and intestinal forms of schistosomiasis are prevalent throughout the country with geographically varying degrees of prevalence [11], [12], [13], [14]. The most recent survey in 2004–2006 showed a prevalence of 38.3% (ranging 0.0–99.0%) for *S. haematobium* and 6.7% (ranging 0.0–94.9%) for *S. mansoni* [14]. Mali was one of the first countries in sub-Saharan Africa to initiate a national schistosomiasis control programme. Control efforts were initiated in the Bandiagara district (Mopti region) as a component of a dam-building project in 1978 and it became a national programme in 1982 [11], [14]. The initial control program with PZQ distribution was implemented by the Ministry of Health, through the National Institute of Public Health Research (INRSP), in collaboration with WHO and with funds from the German Technical Co-operation (GTZ) [11]. GTZ's support for the control programme ceased in 1992 and the Malian government took over the financial responsibilities. However, lack of resources led to control activities being considerably reduced.

In 2004, a new initiative to resume the national control activities was set up with technical and financial support from the Schistosomiasis Control Initiative (SCI) [15], [16]. Mass drug administration (MDA) with PZQ commenced in 2005 in four endemic regions, targeting only school-age children (7–14 years old) attending schools and in 2006 in two other regions, targeting all school-age children (5–15 years old) [16]. In 2007, the schistosomiasis control programme became part of the integrated national control programme on NTDs, funded primarily by the United States Agency for International Development (USAID) NTD Control Program managed by RTI International and implemented initially by International Trachoma Initiative (2007) and latterly by Helen Keller International (since 2008) [17]. Since then, MDA with PZQ has been steadily scaled up to cover seven regions plus Bamako, targeting all school-age children and adults at high risk in hyper-endemic regions, achieving 100% geographical coverage and 72–100% programme coverage [17].

To monitor the progress and impact of the national schistosomiasis control programme, limited parasitological surveys were conducted in selected sentinel schools. The current paper presents the parasitological impact of repeated treatment on schistosomiasis in the highly endemic region of Segou and the recommendations for future control efforts.

## Methods

### Ethical clearance

Mali's schistosomiasis control programme is a national disease control programme using the WHO endorsed preventive chemotherapy strategy [5], and therefore MDA with praziquantel did not require specific ethical clearance. Data collection at sentinel sites was within the framework of the national control programme and was approved by the Ministry of Health. Before doing the survey at each sentinel school, informed verbal consent was first obtained from the chief of the village and the parents of children during the village meeting and was also obtained from school teachers at schools prior to the recruitment of children. These were recorded by the survey team leader. During recruiting, informed verbal consent was obtained from each child with the presence of school teachers. Any children who did not want to participate were free to leave. In Mali, for traditional cultures and low literacy rate in villagers, verbal consent was deemed accepted procedures and approved by the Ministry of Health.

### Mass drug administration

Mali's national schistosomiasis control programme adopts the preventive chemotherapy strategies recommended by WHO [4], [5]. An MDA campaign is designed each year and health personnel at regional, district and community health centres are mobilized. Drugs are distributed through the school-based delivery in schools targeting school-going children and through the community-based delivery in villages targeting non-school-going children and community members at risk. School-based drug delivery is carried out by trained school teachers. Community-based drug delivery is carried out by trained community drug distributors. PZQ tablets (600 mg) were delivered using the WHO dose pole method to determine the dosage for each child [18].

### Study sites and survey design

**Figure 1:**
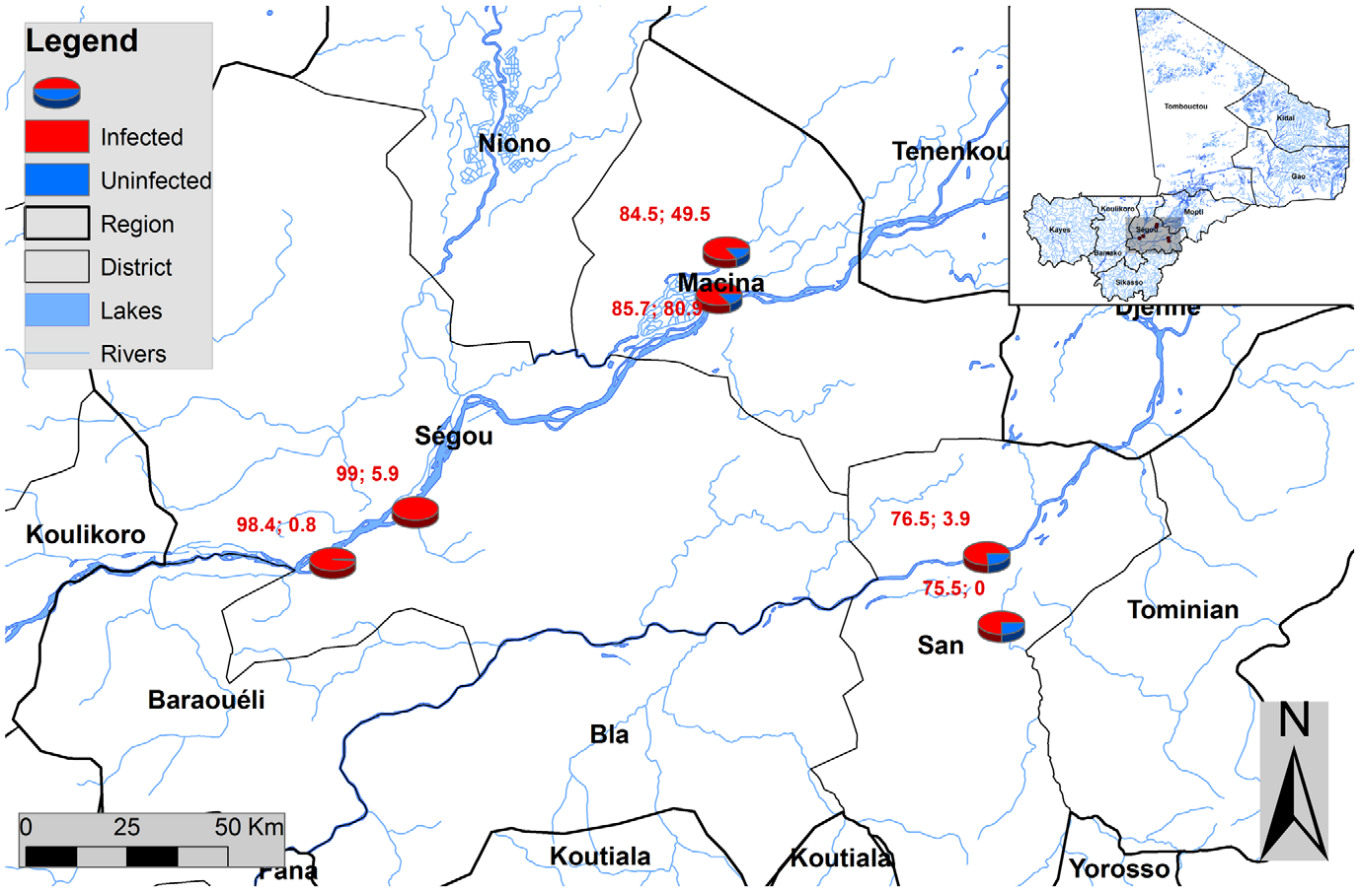
Geographical locations of survey sites in three districts in Segou region, Mali. The figures shown for each survey site in the map are the baseline prevalence of *S. haematobium* (the former) and *S. mansoni* (the latter).

At the start of the national control programme, a national mapping of schistosomiasis was conducted [14]. A number of sentinel sites were randomly selected across the country and the details of the overall study design have been described elsewhere [19], [20]. Baseline data collection before MDA and longitudinal cohort follow-up surveys after MDA were conducted during 2004–2006 [19], [20]. Such longitudinal follow-up surveys stopped after 2006 due to cessation of the SCI funding. In 2010, further cross-sectional survey was undertaken in three health districts (Segou, Macina, and San) in the Segou region, which is highly endemic with schistosomiasis according to the previous mapping surveys [12], [13], [14]. MDA in this region started in 2005. Before the survey in 2010, Segou and Macina districts received four rounds of MDA in 2005, 2006, 2008 and 2009, while San district received three rounds of MDA in 2005, 2006 and 2008. Segou district is located on the Niger River basin; Macina district is an irrigation system area for rice cultivation; and San district reflects a Sahelian environment. The main activities of the population are agriculture, with predominantly rice cultivation and vegetable growing (Macina) and cultivation of millet (San, Segou). The three districts were selected based on their ecological transmission profiles, thus the sentinel sites represent different transmission settings. Two sentinel sites/schools were selected from each district. A total of six sites were surveyed in 2010. The location and the baseline prevalence of schistosomiasis for these six sites are shown in Figure 1. The sampling method for children of 7–14 years old within each school was similar to that used in the baseline survey in 2004 [19], [20]. Briefly, within each site/school, 14 children (7 males and 7 females) were randomly selected from each of eight age groups of 7–14 years, giving a total number of approximately 110 children per school.

### Parasitological examination

#### Urine filtration

From each child, two urine specimens were collected on two consecutive days to determine the prevalence and intensity of *S. haematobium* infection using the filtration method. 10 ml of urine was passed through a Whatman filter paper (Ø =  25 mm) (Brentford, United Kingdom) using a Millipore Swinnex filter holder (Billerica, MA). Filters were stained with 3% ninhydrin and microscopically examined for *S. haematobium* eggs. The intensity of *S haematobium* infection was expressed as number of eggs per 10 ml of urine and the mean intensity of infection was the arithmetic mean of egg count in the two urines samples.

#### Stool examination

One single stool sample from each child was collected and examined with the standard Kato-Katz method. Duplicate Kato-Katz slides were prepared from each sample and examined on the same day. Individual egg output was expressed as eggs per gram of faeces (epg) which was calculated as the arithmetic mean of the two individual slide counts, multiplied by 24.

### Data analysis

Cross-sectional analysis was conducted on the baseline data collected in 2004 before MDA and the data collected in 2010 after a number of rounds of MDA. The 7-year olds were the first year pupils in the schools and these children were supposed to be treated by community-based drug delivery before they joined the schools. The infection status in this group of children would represent the quality of treatment in the community [21]. Therefore, we also compared the data in the 7-year olds only from the baseline (2004), after one treatment (2005) and after 3–4 rounds of treatments (2010). Prevalence and intensity of infection with 95% confidence intervals were calculated using the SPSS (IBM, version 19) Complex Samples module taking into consideration the cluster nature of school children using district as strata and school as clusters. Samples were weighted according to the population sizes in the surveyed districts when calculating the overall prevalence and intensity of infection. Arithmetic mean intensity of infections from all the subjects examined (including both positive and negative) was used in the analysis [22], [23]. The individual infection was categorized as heavy (≥50 eggs/10 ml of urine) and light (<50 egg/10 ml of urine) infections for *S. haematobium*, and heavy (≥400 epg), moderate (100–399 epg) and light (1–99 epg) infections for *S. mansoni*. The Chi-squared test was used to compare differences in prevalence and the Kruskal–Wallis test was used to compare differences in mean intensities. The coordinates of each survey site was identified on Google maps and the site location map was drawn in ArcMap version 10 (ESRI, Redlands, CA).

### Mathematical modelling of the expected outcomes

The expected prevalence and intensity of infection in 2010 was predicted by mathematical models. The dynamic schistosomiasis transmission model, EpiSchisto, predicts the impact of chemotherapy and has been validated for *S. haematobium* and for *S. mansoni* [24], [25]. For easier manipulation, the equations in EpiSchisto were coded into the Berkeley Madonna modelling software (version 5.0) [26].

Schistosomiasis infection typically displays a high degree of overdispersion (or aggregation, whereby the majority of the parasites are harboured in a minority of human hosts) and the degree of this overdispersion often varies significantly between locations and between species. The distribution of parasites amongst hosts is often taken to be adequately approximated by the negative binomial distribution [27], which is described by two parameters: m, the mean intensity of infection, and *k*, the inverse of the overdispersion. In the EpiSchisto software, the overdispersion parameter *k* is allowed to vary linearly with the mean intensity of infection via the following relationship:


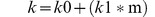
(1)

Given the high levels of heterogeneity observed in schistosomes in different epidemiological settings, the *k* values were estimated separately for each of the three districts (Macina, San, Segou) and for each of the two species (Table 1). The parameters were fitted using a maximum likelihood estimation approach [28]. The observed baseline mean intensity of infection (m) and the estimated *k* values were input into the models for prediction, using the treatment schedules in each district. Treatment was assumed to have 95% efficacy [24], [29], and we used 75% as the minimum treatment coverage required as per WHO recommendations each year for each district.

**Table 1:** Estimated *k* values for each district and for each species^a^.

Species	District	*k0*	*k1*
*Schistosoma haematobium*	Macina	0.25640	0.00131
	San	0.09785	0.0344
	Segou	0.37688	0.00096
*Schistosoma mansoni*	Macina	0.08098	0.00017

a *k* values for *S. mansoni* in San and Segou districts were not estimated due to the very low prevalence.

## Results

### Comparison of the data in 2010 with the baseline in 2004

Table 2 summarizes the prevalence and the intensity of infection in school children from the baseline in 2004 and the survey in 2010. The data collected from 640 school children aged 7–14 years in 2010 from six sentinel schools were compared with the baseline data from the same age groups (648 school children) in the original cohort of the same schools.

**Table 2:** Prevalence and mean intensity of infection (95% CI) of schistosomiasis in 2004 and 2010.

	No examined		*S. haematobium*		*S. mansoni*	
	2004	2010	2004	2010	2004	2010
**Prevelence (%)**						
Overall	648	640	88.0 (85.4–90.4)	61.7 (57.9–65.4)	17.3 (14.6–20.4)	12.7 (10.3–15.6)
Segou	224	221	98.7 (97.9–99.5)	87.8 (83.6–91.2)	3.1 (1.6–5.8)	5.4 (3.4–8.8)
Macina	212	213	84.0 (77.2–88.9)	55.9 (47.4–63.3)	66.5 (58.5–73.5)	47.7 (39.2–55.8)
San	212	206	75.9 (69.6–81.2)	29.1 (23.5–35.8)	1.9 (0.8–4.9)	0 (0–1.8)
By Sex						
Boys	326	336	88.3 (84.3–91.3)	63.6 (58.2–68.5)	17.8 (14.1–22.4)	15.5 (12.0–19.9)
Girls	322	304	87.7 (83.9–91.0)	59.7 (54.2–65.1)	16.9 (13.3–21.5)	9.7 (6.8–13.5)
**Intensity of infection**						
Overall	648	640	180.4 (110.1–250.7)	33.2 (0–71.6)	88.2 (0–296.7)	43.2 (0–172.8)
Segou	224	221	325.7 (234.2–417.1)	67.4 (0–151.9)	4.7 (0–20.7)	3.5 (0–14.5)
Macina	212	213	75.8 (8.7–142.9)	6.3 (3.0–9.7)	372.1 (0–1198.0)	194.4 (0–757.5)
San	212	206	51.2 (0–147.4)	4.1 (2.6–5.6)	1.2 (0–5.4)	0 (0–0)
By Sex						
Boys	326	336	191.8 (95.9–287.7)	37.5 (0.8–74.1)	121.8 (0–419.6)	55.3 (0–217.9)
Girls	322	304	169.0 (125.7–212.3)	28.6 (0–68.6)	54.4 (0–176.5)	30.1 (0–123.4)

Note: 1. Intensity of infection: eggs/10 ml of urine for *S. haematobium* and epg for *S. mansoni*. 2. Segou and Macina districts received four rounds of MDA in 2005, 2006, 2008 and 2009, while San district received three rounds of MDA in 2005, 2006 and 2008.

In 2004, *S. haematobium* infection was extremely prevalent (75.9–98.7%) and heavy (mean intensity of infection over 50 eggs/10 ml of urine) in all three districts surveyed. Macina district also had heavy *S. mansoni* infection (prevalence 66.5% and mean intensity of infection 372.1 epg). Overall prevalence of *S. haematobium* infection was 88% and that of *S. mansoni* infection was 17.3%.

In 2010, overall prevalence of *S. haematobium* infection was 61.7%, a significant reduction of 30% from the baseline in 2004 (p<0.01), while overall prevalence of *S. mansoni* infection was 12.7% which was not significantly different from the baseline in 2004 (p>0.05). Overall mean intensity of *S. haematobium* and *S. mansoni* infection was 180.4 eggs/10 ml of urine and 88.2 epg in 2004 respectively. These were reduced to 33.2 eggs/10 ml of urine and 43.2 epg in 2010 respectively, significant reductions of 81.6% and 51% (p<0.0001). Among three districts, different degrees of reduction were seen in *S. haematobium* infection in 2010, with San showing 61.7% and 92.0%, Macina 33.5% and 91.7% and Segou 11.0% and 79.3% in prevalence and intensity of infection respectively.

There were no statistically significant differences between boys and girls in prevalence and mean intensity of *S. haematobium* infection either in 2004 or in 2010 and of *S. mansoni* infection in 2004 (p>0.05). However, there was a significant difference between boys and girls in prevalence and mean intensity of *S. mansoni* infection in 2010 (p<0.05). As between boys and girls, similar pictures were seen among different ages in the prevalence and the mean intensity of *S. haematobium* or *S. mansoni* infection in 2004 and 2010 (details not shown).

**Figure 2:**
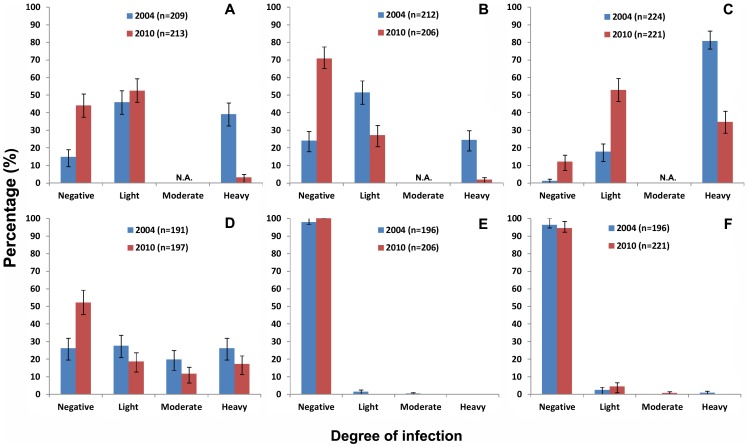
Percentage proportion of light, moderate or heavy infections in 2004 and 2010. Upper panel: *S. haematobium* infection in Macina (A), San (B) and Segou (C), categorized as light (<50 eggs/10 ml of urine) and heavy (≥50 eggs/10 ml of urine) infections. Lower panel: *S. mansoni* infection in Macina (D), San (E) and Segou (F), categorized as light (1–99 epg), moderate (100–399 epg) and heavy (≥400 epg) infections. N.A.: not applicable. Error bars represent the 95% CI.

The overall proportion of heavy *S. haematobium* infections was reduced from 48.8% in 2004 to 13.8% in 2010, and the overall proportion of moderate and heavy *S. mansoni* infection was reduced from 15.6% in 2004 to 9.4% in 2010, both significantly (p<0.01). The shift of the categories of intensity of infection in three districts is illustrated in Figure 2.

### Comparison of the observed results with the expected impact through mathematical modelling

**Figure 3:**
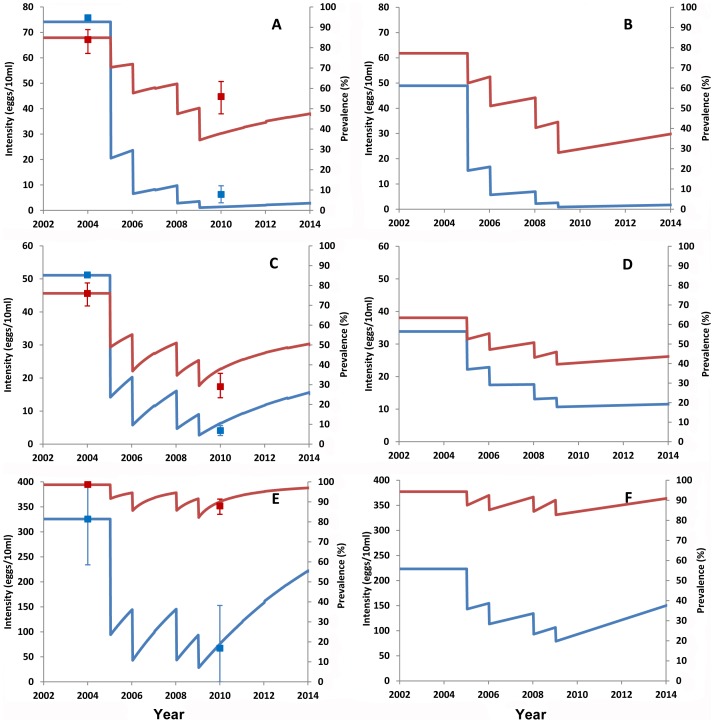
Comparison of the observed *S. haematobium* prevalence and intensity with the predicted changes. Prevalence is shown in red and intensity of infection is shown in blue. *S. haematobium* infection is shown in 7–14 year group (A, C and E) and in entire population (B, D and F). A and B: Macina district; B and C: San district; and E and F: Segou district. Lines show the predicted changes and squares in A, C and E show the observed results. Error bars represent the 95% CI.

**Figure 4:**
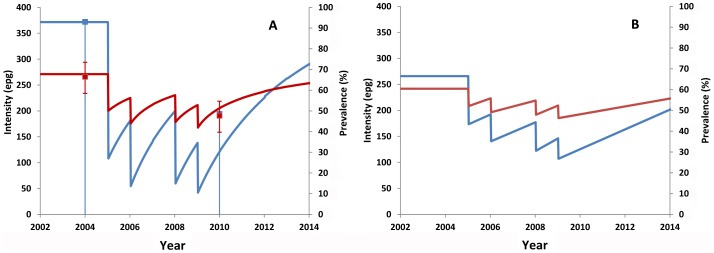
Comparison of the observed *S. mansoni* prevalence and intensity with the predicted changes in Macina. Prevalence is shown in red and intensity of infection is shown in blue. *S. mansoni* infection is shown in 7–14 year group (A) and in entire population (B). Lines show the predicted changes and squares in A show the observed results. Error bars represent the 95% CI.

The prevalence and intensity of infection observed in 2010 were compared to those predicted from mathematical models. The model-derived changes in the prevalence and intensity of infection were estimated for two separate groups: the 7–14 years old and the entire population. The predicted results of the 7–14 year group allow for direct comparison with the observed results, while the predicted results in the wider age range (0–60 years) indicate the expected treatment impact in the entire communities. The results are presented in Figure 3 for *S. haematobium* and in Figure 4 for *S. mansoni* in Macina district. *S. manosni* in the other two districts was not analyzed using mathematical models as the prevalence was very low. Overall, the predicted changes in the prevalence and intensity of infection were within or close to the 95% confidence intervals of the observed results in each district. This suggests that the observed results in each district were largely expected using the current MDA strategy.

### Comparison of the data in children of 7 years old in 2004, 2005 and 2010

As shown in Table 3, *S. haematobium* prevalence in 2010 was 59.4%, significantly lower than 90.5% in 2004 (p<0.001) but higher than 47.2% in 2005 (p<0.05), with an overall reduction of 34.4%. *S. haematobium* mean intensity of infection was 25.6 eggs/10 ml of urine in 2010, significantly lower than 194.9 eggs/10 ml of urine in 2004 (p<0.001) but not significantly different from 38.0 eggs/10 ml of urine in 2005 (p>0.05). There was a significant overall reduction of 86.9% in *S. haematobium* mean intensity of infection.

**Table 3:** Prevalence and mean intensity of infection (95% CI) in the 7-years old.

Year	2004	2005	2010
***S. haematobium***			
No of children examined	137	195	143
Prevalence (%)	90.5 (84.4–94.4)	47.2 (40.3–54.2)	59.4 (51.3–67.1)
Intensity of infection (eggs/10 ml of urine)	194.9 (142.8–247.0)	38.0 (19.1–56.8)	25.6 (8.3–42.8)
***S. mansoni***			
No of children examined	124	194	136
Prevalence (%)	20.2 (14.1–28.1)	16.0 (11.5–21.8)	26.5 (19.8–34.5)
Intensity of infection (epg)	85.0 (30.7–139.4)	49.5 (6.7–92.3)	71.7 (35.5–107.8)

*S. mansoni* prevalence was 26.5% in 2010, not significantly different from 20.2% in 2004 (p>0.05) but significantly higher than 16.0% in 2005 (p<0.05). *S. mansoni* mean intensity of infection was 71.7 epg in 2010, not significantly different from 85.0 epg in 2004 (p>0.05) but higher than 49.5 epg in 2005 (p<0.05). There was no significant overall change in either prevalence or mean intensity of *S. mansoni* infection.

## Discussion

The baseline data showed that three districts surveyed in the Segou region were highly endemic with schistosomiasis. These three districts represent three different epidemiological settings: river basin, irrigated rice field and Sahelian area. Two sites in Segou district along the Niger River showed the highest level of infection and school children were nearly universally infected with *S. haematobium*; two sites in Macina district in the irrigation area showed high level of infection with both *S. haematobium* and *S. mansoni*; and two sites in San district in the Sahelian area showed lower but still high level of infection of *S. haematobium* (Figure 1). This represents significant disease burden caused by schistosomiasis in children in this region.

Before the survey in 2010, schoolchildren in Segou and Macina districts received four rounds of treatment and those in San district received three rounds of treatment. And yet the overall prevalence of *S. haematobium* infection in 2010 remained unacceptably high, though a significant 30% reduction compared with the baseline, while overall prevalence of *S. mansoni* infection did not show a significant reduction. Looking at three epidemiological settings, *S. haematobium* prevalence at two sites along the Niger River was almost at the baseline level; prevalence in the irrigation area showed a modest reduction (around 30%) for both *S. haematobium* and *S. mansoni*; while *S. haematobium* prevalence in the Sahelian area showed the biggest drop (61.7%). Mathematical modelling of the changes in prevalence and intensity of infection in three districts showed that the observed results were in line with the expected changes in each of three epidemiological settings. Such varying level of reduction in different epidemiological settings may be explained by different transmission levels and patterns in these settings: permanent transmission along the big river versus seasonal transmission in temporary water bodies. In the Sahelian environment in Burkina Faso and Niger, one round of MDA significantly reduced *S. haematobium* prevalence to a very low level, which remained low for 2–3 years [21], [30], [31]. However, along the Niger River in Niger, a similar situation as in Segou district, one year after treatment the *S. haematobium* prevalence bounced back to nearly the pre-treatment level [32], suggesting high level of transmission and frequent water contact activities in such locations. In Ugandan national control programme, *S. mansoni* prevalence also showed less reduction in highly endemic areas along the lake shores after repeated MDA [33].

Regardless of persistent prevalence, significant reduction in overall intensity of infection was seen in 2010 for both *S. haematobium* (by 81.6%) and *S. mansoni* (by 51%). The proportion of heavy *S. haematobium* infection was reduced from 48.8% to 13.8%, and the proportion of moderate and heavy *S. mansoni* infection was reduced from 15.6% to 9.4%. This is important as it is well known that the severity of morbidity caused by schistosomiasis is closely related to the intensity of infection. The heavier infections, in general, cause the more severe morbidity [34]. It is therefore anticipated that significant morbidity would have been prevented or reverted due to MDA in the national control programme. This is in line with what was achieved in other national MDA programmes in both East and West Africa through preventive chemotherapy [8], [9], [21], [33], [35]. However, there still exist a significant proportion of children with relatively low intensity of infections: 27.2–52.9% with *S. haematobium* and 0–18.8% with *S. mansoni* in three districts as shown in Figure 2, not including those undetected due to the low sensitivity of the diagnostic techniques. Such light infections have long been overlooked in terms of the morbidity consequences, and recent findings suggest that light infections can cause considerable morbidity due to anaemia, chronic pain, diarrhoea, exercise intolerance and undernutrition [34], [36]. The objective was to reduce morbidity due to schistosomiasis by regular treatment according to the WHO recommendations [4], [5], and regular MDA in the highly endemic regions in Mali may have just served this purpose, however, the persistent infection in the Segou region highlights the need for a persistent effort.

Schistosomiasis is one of the five major NTDs currently targeted for preventive chemotherapy through the integrated MDA strategy [5]. WHO published its first NTD report in 2010 [37], and has just launched a new roadmap for overcoming the burden of the NTDs, including elimination of schistosomiasis [38]. To achieve the objectives, comprehensive control measures are recommended including preventive chemotherapy, intensified case management, vector and intermediate host control, veterinary public health at the human-animal interface, and provision of safe water, sanitation and hygiene [37], [38]. However, in the current schistosomiasis control in the integrated national NTD control programmes, such as the one in Mali, the funding focus has been almost exclusively on preventive chemotherapy, while other components such as case management, snail control, and safe water, sanitation and hygiene are not implemented due to lack of funding. The current results, together with others, suggest that MDA alone in such highly endemic areas is simply not enough. In sub-Saharan countries, such as Mali, it is almost impossible to implement such other components in schistosomiasis control programme without proper external funding. It is therefore understandable that schistosomiasis prevalence persists in such high transmission areas even after several rounds of MDA. To achieve the said objectives in the new WHO roadmap towards schistosomiasis elimination [38], funds must be made available for the national programmes to implement the recommended comprehensive control measures.

Persistent prevalence of *S. haematobium* and *S. mansoni* infections and relatively high level of intensity of infection for *S. mansoni* (in the highly endemic Macina district) after several rounds of MDA indeed raised some concerns in schistosomiasis control in Mali. As shown in the 7-year olds, the first MDA reduced both prevalence and intensity of infection; however, prevalence for both species and intensity for *S. mansoni* had significantly rebounded since 2005. One concern is the treatment coverage and quality in Segou region. The reported program coverage in Segou region for schistosomiasis treatment was 56.4% in 2005, 75.1% in 2006, 0% in 2007, 76.4% in 2008 and 71.6% in 2009 according to the national schistosomiasis control programme. However, a post-MDA survey conducted in 2009 showed a significantly lower coverage than the reported coverage (details not shown). Lessons have been learned and measures have since been taken to increase the drug coverage. Another concern may be the possible drug tolerance, particularly for *S. mansoni*. PZQ has been used at large scale in Mali for rather a long time, first between 1982 and 1992 and second since 2005. Mounting drug pressure may have caused parasites to establish a certain level of tolerance. A similar situation was observed in a recent treatment trial in Niger [32]. These together suggest that a closer monitoring of drug efficacy in the national MDA programme is required.

It is noted that the current results are limited due to lack of more sentinel site surveys and only represent areas with similarly high level of transmission as in Segou region. Schistosomiasis is endemic in all regions of Mali with varying levels of endemicity, and the current situation in other regions is not clear. After several rounds of MDA, there is a need for a national survey in order to monitor the treatment impact, adjust the treatment focus and increase cost-efficiency of control measures.

### Conclusion

MDA with PZQ has been conducted in Mali since 2005 and several rounds of treatment have been delivered. Sentinel site surveys in Segou region showed that significant reduction in intensity of infection on both infections and modest but significant reduction in *S. haematobium* prevalence were achieved in highly endemic regions. Most importantly, proportion of moderate and heavy infections was reduced in school-age children. Significant prevention and reversion of morbidity due to schistosomiasis was anticipated in the Mali NTD control programme. However, persistent prevalence of both infections and relatively high level of intensity of *S. mansoni* infection suggests that more intensified control measures be implemented, possibly more frequent treatments, improved coverage and improved water and sanitation. In addition, closer monitoring and evaluation activities are needed in the programme to monitor the drug tolerance and to adjust treatment focus.
